# Adolescent Maturation of Dopamine D1 and D2 Receptor Function and Interactions in Rodents

**DOI:** 10.1371/journal.pone.0146966

**Published:** 2016-01-19

**Authors:** Jennifer B. Dwyer, Frances M. Leslie

**Affiliations:** Department of Pharmacology, University of California, Irvine, Irvine, California, United States of America; Brock University, CANADA

## Abstract

Adolescence is a developmental period characterized by heightened vulnerability to illicit drug use and the onset of neuropsychiatric disorders. These clinical phenomena likely share common neurobiological substrates, as mesocorticolimbic dopamine systems actively mature during this period. Whereas prior studies have examined age-dependent changes in dopamine receptor binding, there have been fewer functional analyses. The aim of the present study was therefore to determine whether the functional consequences of D1 and D2-like activation are age-dependent. Adolescent and adult rats were given direct D1 and D2 agonists, alone and in combination. Locomotor and stereotypic behaviors were measured, and brains were collected for analysis of mRNA expression for the immediate early genes (IEGs), *cfos* and *arc*. Adolescents showed enhanced D2-like receptor control of locomotor and repetitive behaviors, which transitioned to dominant D1-like mechanisms in adulthood. When low doses of agonists were co-administered, adults showed supra-additive behavioral responses to D1/D2 combinations, whereas adolescents did not, which may suggest age differences in D1/D2 synergy. D1/D2-stimulated *IEG* expression was particularly prominent in the bed nucleus of the stria terminalis (BNST). Given the BNST’s function as an integrator of corticostriatal, hippocampal, and stress-related circuitry, and the importance of neural network dynamics in producing behavior, an exploratory functional network analysis of regional IEG expression was performed. This data-driven analysis demonstrated similar developmental trajectories as those described in humans and suggested that dopaminergic drugs alter forebrain coordinated gene expression age dependently. D1/D2 recruitment of stress nuclei into functional networks was associated with low behavioral output in adolescents. Network analysis presents a novel tool to assess pharmacological action, and highlights critical developmental changes in functional neural circuitry. Immature D1/D2 interactions in adolescents may underlie their unique responses to drugs of abuse and vulnerability to psychopathology. These data highlight the need for age-specific pharmacotherapy design and clinical application in adolescence.

## Introduction

Adolescence is a transitional developmental period between childhood and adulthood, during which drug abuse often begins [1], and psychopathologies emerge or change symptomology [2]. These unique clinical features are thought to be mediated by changes in the structural [3], functional [4], and neurochemical [5] organization of the brain. The dopamine (DA) system undergoes striking maturation during adolescence [6], which has significant implications for adolescent-onset drug abuse and psychiatric disorders. Consistent with developmental changes in DA signaling, adolescents respond uniquely to dopaminergic drugs. Teenagers exhibit blunted behavioral responses to indirect DA agonists like cocaine and amphetamine [7], but exaggerated responses to DA receptor antagonism [8].

Rodent adolescents (conservatively estimated at postnatal day (P) 28–42) are appropriate animal models, as they share evolutionarily conserved behaviors such as risk-taking, novelty seeking, and peer association, and display many of the same patterns of structural and neurochemical brain maturation as humans [9]. While the consensus definition of adolescence in the rodent continues to evolve in light of emerging behavioral, endocrine, and imaging data [10,11], adolescent animals show unique responses to drugs that target the DA system, including blunted cocaine-induced locomotion [12], stereotypy [13], and sensitization [14]. Cocaine also induces unique patterns of neural activation in adolescents, as measured by immediate early gene (IEG) expression [15,16] suggesting that the neural circuitry underlying behavioral responses is immature.

The effects of cocaine are predominantly mediated by the prolonged action of DA at D1-like and D2-like receptors. Although initially categorized by the ability to activate (D1-like) or inhibit (D2-like) adenylyl cyclase, these receptors have since been shown to couple to multiple signaling pathways [17]. D1 receptors not only couple to Gs proteins (D1[Gs]) to stimulate adenylyl cyclase, but also to Gq proteins (D1[Gq]) which activate phospholipase C and intracellular Ca^++^ release [18], and may have differential interactions with D2-like receptors [19]. While most D1-like agonists have some efficacy at D1[Gs] and D1[Gq], second messenger-selective agonists have been developed [20]. Although few studies have assessed second-messenger selective signaling in adolescence [21], DA receptor expression is known to be age-dependent, with transient overproduction and subsequent pruning of D1 and D2 binding sites in the adolescent prefrontal cortex [6,22] and striatum [23,24].

Although age differences in the functional consequences of receptor activation are less well-studied, rodent behavioral and imaging studies suggest that adolescent D1 receptors are hypofunctional and D2 receptors are hyperfunctional [25,26]. However, it is the synergistic interaction of combined D1/D2 stimulation that is thought to mediate behavioral responses and corticostriatal IEG expression in adults [27,28], although significant controversy remains regarding whether molecular versus circuit level mechanisms are predominantly at play [29]. While there have been few dedicated evaluations of the ontogeny of this phenomenon [30,31], molecular evidence suggests that D1/D2 synergy is immature in adolescence [19], which could underlie blunted behavioral responses to indirect DA agonists. The present study uses behavioral, neurochemical, and functional network approaches to test the hypothesis that functional D1/D2 interactions are immature during adolescence. The following data suggest that D1/D2 synergy may not be a fundamental feature of adolescent locomotor control and corticostriatial engagement as it is in adults. Adaptation of network analysis generates a further hypothesis that D1/D2-mediated recruitment of stress nuclei into functional networks inhibits locomotor activity in adolescence.

## Materials and Methods

### Materials

Quinpirole hydrocloride (Tocris Bioscience, Ellisville, MO) was dissolved in sterile saline. SKF83959 (Sigma Aldrich, St. Louis, MO) and SKF83822 (NIMH Chemical Synthesis Program, Bethesda, MD) were initially dissolved in 10% DMSO and diluted in sterile saline. For in situ hybridization, the following materials were used: poly-L-lysine, RNaseA, restriction enzymes, T3, T7 polymerases, proteinase K and yeast tRNA (Boehringer Mannheim Biochemicals, Indianapolis, IN); formamide (Fluka, Ronkonkoma, NY); dextran sulfate (Pharmacia, Piscataway, NJ); Hyperfilm, Bmax (Amersham, Arlington Heights, IL); ^35^S-Uridine triphosphate (^35^S-UTP) (specific activity: 20–40 Ci/mmol) (Perkin Elmer).

### Animals

Male Sprague—Dawley rats (Charles River, Wilmington, MA) were group housed in a temperature (21°C) and humidity (50%) controlled room on a 12h light—dark cycle (lights on 0700–1900), with unlimited access to food and water. Adolescents, aged P32 on the experimental day, and adults, aged P90, were habituated to the vivarium and handled for 5 days before use. Each animal participated in one experiment, receiving a single drug and dose. Experiments were carried out in accordance with, and were explicitly approved by the Institutional Animal Care and Use Committee (IACUC) at the University of California, Irvine, and were consistent with federal guidelines.

### Behavior (Locomotion and Stereotypy)

#### Experiment 1

To assess age differences in the behavioral sensitivity to D1 and D2-like agonists, dose response curves were constructed in adolescents and adults. Animals were assessed for locomotor and stereotypic behavior following the administration of the D1[Gs]-selective agonist, SKF83822 [32], the D1[Gq]-selective agonist, SKF83959 [33], or the D2-like agonist, quinpirole [34] (see S1 File, Supplemental Methods).

#### Experiment 2

To examine D1/D2 interactions at the behavioral level, locomotion and stereotypy induced by low-dose combinations of quinpirole and either SKF83822 or SKF83959 were assessed. While arguably the most thorough way to assess drug interactions entails testing multiple D1/D2 combinations corresponding to several different points of efficacy along the dose response curves [35], conducting such a behavioral analysis at two age points would be difficult to power within ethical standards of animal use. Thus, the simplified approach of Johnstone et al.[36] was chosen as one way to evaluate D1/D2 interactions, by assessing combinations of low agonist doses (chosen based on the dose response curves from Experiment 1), which did not significantly increase locomotor behavior relative to saline on their own. The goal of these studies was to evaluate whether D1/D2 combinations at half-doses are able to produce behavior that additive doses of either agonist alone are unable to stimulate. Synergistic or supra-additive D1/D2 interactions were assessed by comparing D1/D2 combination doses with additive doses of each agonist alone, as described previously [36]. Adolescent and adult rats were randomly assigned to one of 6 treatment groups: saline, quinpirole (0.4mg/kg), SKF83822 (0.06mg/kg), SKF83959 (0.6mg/kg), quinpirole (0.2mg/kg) + SKF83822 (0.03mg/kg), or quinpirole (0.2mg/kg) + SKF83959 (0.3mg/kg). Following behavioral testing (i.e. 30 min following drug injection, chosen as *cfos* and cytoplasmic *arc* mRNA expression peaks 30 minutes post-stimulus [37,38]), animals were sacrificed via rapid decapitation, and brains were collected, frozen in -20°C isopentane, and stored at -80°C until use for *in situ* hybridization.

### *In Situ* Hybridization

Twenty μm coronal brain sections were cut and processed for *cfos* and *arc in situ* hybridization and were analyzed quantitatively via computer-based image analysis (MCID, Image Research Inc., St Catharines, ON, Canada) [39] (see also S1 File, Supplemental Methods). Levels of mRNA expression were determined in *a priori* regions of interest, based on their expression of D1 and D2 receptors, their known roles in DA-mediated behaviors, and their expression of both *cfos* and *arc*: *prefrontal cortex* (cingulate (Cg1), prelimbic (PrL), infralimbic (IL), ventrolateral/orbital (VLO)); *sensorimotor cortex* (primary motor (M1), secondary motor (M2), primary sensory (S1), caudal primary motor (cM1), agranular insular (AI), caudal agranular insular (cAI)); *striatum* (dorsomedial caudate putamen (dmCPu), dorsolateral caudate putamen (dlCPu), ventromedial caudate putamen (vmCPu), ventrolateral caudate putamen (vlCPu)), nucleus accumbens core (NAcC), nucleus accumbens shell (NAcSh)); *hippocampus* (CA1, CA2, CA3, dentate gyrus (DG), medial septum (MS), lateral septum (LS)); and *amygdala/hypothalamus* (bed nucleus of the stria terminalis (BNST), paraventricular nucleus of the hypothalamus (PVN), basolateral amygdala (BLA), central nucleus of the amygdala (CeA), medial nucleus of the amygdala (MeA)).

### Data Analysis

#### Behavioral data

Locomotion and stereotypy were analyzed separately for each drug. Initial analyses of 30 min totals of drug-induced activity included 2-way ANOVA, with age and drug dose as dependent variables for dose-response data, and age and drug for combination data. Following a significant effect or interaction of age, adolescents and adults were analyzed separately via 1-way ANOVA, with Dunnett or Bonferonni post-hoc tests.

While there is some debate regarding the parametric analysis of stereotypic data [40], the scale used in the present study [41] has traditionally been analyzed parametrically. This scale largely measures intensity of behavior, rather than mere counting of instances of behavior (see S1 File, Supplemental Methods). Shapiro-Wilk testing of normality revealed that the majority of experimental groups conformed to the assumption of normality, and the ANOVA is a parametric test that is robust to even gross deviations in normality [42].

#### Immediate early gene regional analysis

Analysis of individual regional *cfos* and *arc* mRNA expression consisted of a 2-way ANOVA with age and drug as dependent variables. Following a significant effect of age, or an interaction of age with drug, adolescents and adults were analyzed separately via 1-way ANOVA for drug, using Bonferroni post-hoc corrections to compare all drug doses to each other. If there were no effects of age, adolescents and adults were pooled and analyzed via 1-way ANOVA for drug with Bonferroni post-hoc corrections. As with the behavioral data, IEG responses were considered synergistic if the D1/D2 combination was significantly different from both saline and from the additive doses of either agonist alone.

#### Analysis of cfos and arc coordinated gene expression

In order to assess network-level coordinated gene expression (CGE), we adapted functional network analysis approaches to regional IEG data [43] (see also S1 File, Supplemental Methods). An adjacency matrix was constructed for each gene (*cfos*, *arc*), in each drug condition, for both adolescents and adults, yielding 24 matrices (S1 Fig provides an example). Each weighted, symmetrical matrix was composed of Pearson coefficients (r) derived from the intersubject correlation of IEG expression between each pair of brain regions analyzed (27 x 27 regions). Matrices were then thresholded at p<0.05, setting all non-significant r-values to zero (S2 Fig provides an example). While the pros and cons of liberal versus conservative network thresholds continue to be debated [44], a rather stringent visualization threshold was selected in order to examine the most robust relationships across several drug conditions. Thresholded network matrices were imported into UCINET software and visualized using Netdraw (UCINET 6.0, Analytic Technologies, Lexington, KY). Individual brain regions were displayed as network nodes and were presented in pseudoanatomical space (S3 Fig). The r-values denoting significant statistical associations between two regions (i.e. CGE, analogous to functional connectivity) were displayed as edges or links connecting two nodes. Both positive and negative functional relationships were visualized, with black lines denoting positive r-values and red lines denoting negative r-values.

Using the open-source brain connectivity toolbox of Sporns [43], community structure was determined by assessing modularity of each network (Matlab R2010a, MathWorks, Natick, MA). This function identifies nodes participating in highly interconnected subgroups within the larger network. The color outlining the nodes demonstrates those that belong to the same subcommunities, highlighting functional relationships between areas that may be anatomically distant.

D1/D2-activated adolescent and adult IEG networks were then compared with networks in saline-treated animals at each age. While thresholded matrices were used for visualization purposes, all r-values were included in network comparisons. Fischer r-to-z transformations were applied to improve normality. Drug-induced differences of correlation strength between each regional pair were calculated by dividing the difference between the z scores by the standard error of the difference. P values associated with Z difference scores were corrected using false discovery rate [45] (*q* = 0.35). A liberal threshold was chosen with the priority of minimizing Type II errors, which are exacerbated with conservative thresholds in networks with low signal-to-noise [46], while still providing moderate Type I error correction. Between-network differences were illustrated using UCINET and Netdraw software.

## Results

### Age Differences in Behavioral Responses to D1 and D2-Like Agonists

**Fig 1:**
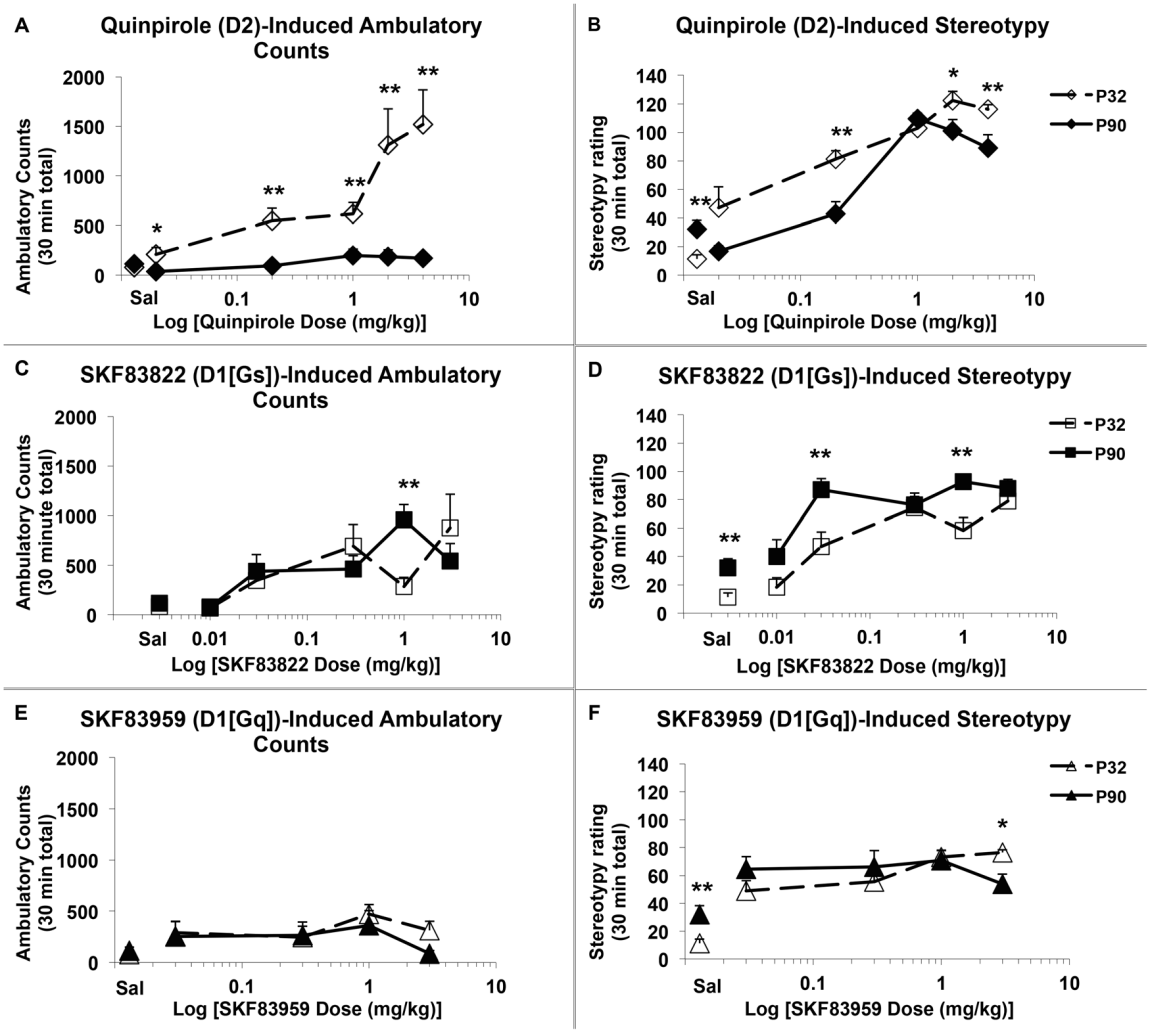
Dose Response Curves for Agonist-Induced Behavioral Response. The D2 agonist, quinpirole, stimulates greater locomotor **(A)** and stereotypic **(B)** behavior in adolescents (P32) than adults (P90). The selective D1[Gs] agonist, SKF83822, stimulates more locomotor **(C)** and stereotypic **(D)** behavior in adults than adolescents. While there are no significant age differences locomotor response to the selective D1[Gq] agonist, SKF83959 **(E)**, 3.0mg/kg SKF83959 stimulates more stereotypy in adolescents than adults **(F)**. Significant age difference, *p<0.05, **p<0.01; n = 6–8 per group.

Significant age differences were observed in behavioral responses to D1 and D2-like agonists (Fig 1). As has been reported previously [25,34], quinpirole-induced locomotion decreased with age. Ambulation (Fig 1A) was influenced by drug dose (F(5,78) = 7.653, p<0.001), age (F(1,78) = 39.226, p<0.001), and the interaction of dose with age (F(5,78) = 5.786, p<0.001), with quinpirole increasing locomotion in adolescents (F(5,41) = 7.631, p<0.001), but not adults (F(5,37) = 1.821, p = 0.133). Stereotypy (Fig 1B) was also influenced by drug dose (F(5,80) = 55.367, p<0.001), age (F(1,80) = 11.192, p = 0.001), and the interaction of dose with age (F(5,80) = 4.967, p = 0.001). While quinpirole increased stereotypy in both adolescents (F(5,42) = 28.087, p<0.001) and adults (F(5,38) = 33.392, p< 0.001), adolescents showed significantly greater stereotypy at several quinpirole doses, despite higher baseline stereotypy in adults.

In contrast, activity induced by the D1[Gs]-selective agonist, SKF83822, increased with age. Locomotion (Fig 1C) showed an effect of drug dose (F(5,89) = 6.811, p<0.001) and a dose x age interaction (F(5,89) = 2.957, p = 0.016). Although SKF83822 increased locomotion in both adolescents (F(5,41) = 3.527, p = 0.010) and adults (F(5,48) = 6.511, p<0.001), adults showed significantly greater ambulatory activity at the 1mg/kg dose. Age (F(1,91) = 20.455, p<0.001) and drug dose (F(5,91) = 21.955, p<0.001) also influenced stereotypy (Fig 1D). While there were drug effects in both adolescents (F(5,42) = 12.312, p<0.001) and adults (F(5,49) = 11.006, p<0.001), stereotypy was significantly higher in adults.

The D1[Gq]-selective agonist, SKF83959, induced low levels of ambulatory activity (Fig 1E), that were influenced by drug dose (F(4,60) = 3.541, p = 0.012), but not age (F(1,60) = 1.233, p = 0.271). In contrast, there was a significant interaction of drug dose with age (F(4,59) = 3.147, p = 0.021) for SKF83959-induced stereotypy (Fig 1F). Although there were drug effects in both adolescents (F(4,29) = 29.708, p<0.001) and adults (F(4,30) = 4.406, p = 0.006), adolescents exhibited higher SKF83959-induced stereotypy at the highest dose tested (p = 0.027).

### Age Differences in Behavioral Responses to D1/D2 Agonist Combinations

**Fig 2:**
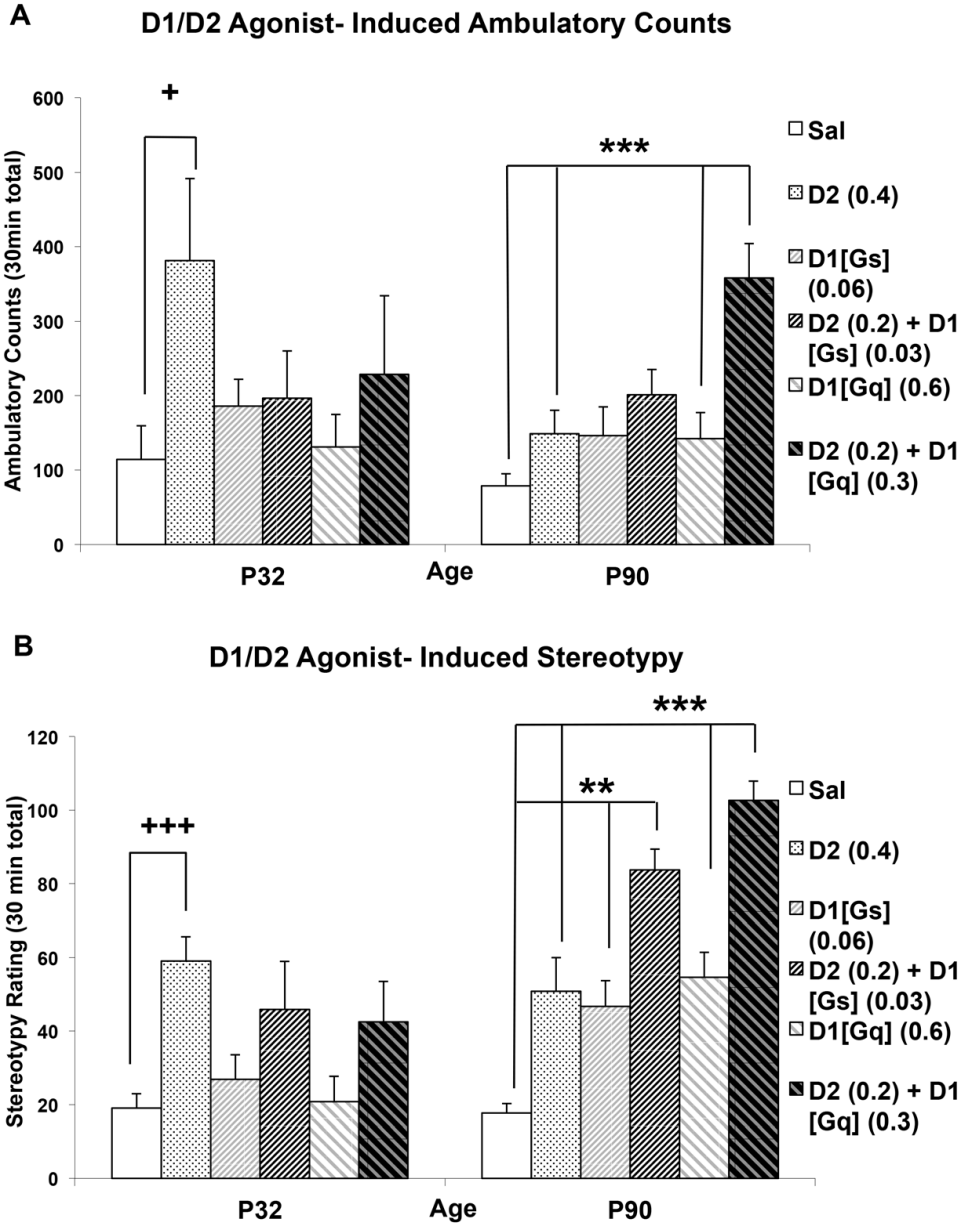
Age Differences in Behavioral Synergy. **(A)** Locomotor and stereotypic response to quinpirole and SKF83822, alone and in combination (D2 (0.2) + D1[Gs]). **(B)** Locomotor and stereotypic response to quinpirole and SKF83959, alone and in combination (D2 (0.2) + D1[Gq]). ***p<0.001 vs. saline and additive doses of each agonist along at the same age. +p<0.05 vs. saline, ++p<0.01; n = 10–19 per group

Behavioral interactions between D1 and D2 receptors were age-dependent, with D1/D2 agonist combinations producing supra-additive responses in adults but not adolescents (Fig 2). Ambulatory response to D1/D2 combinations (Fig 2A) was influenced by drug (F(5,167) = 4.863, p<0.001) and the interaction of drug with age (F(5,167) = 2.659, p = 0.024). There was a strong trend towards a drug effect on adolescent locomotion (F(5,67) = 2.298, p = 0.055), primarily driven by quinpirole, the only treatment significantly different from saline (p = 0.031). Adult locomotion was influenced strongly by drug treatment (F(5,100) = 8.072, p<0.001), with the response to the combination of SKF83959+quinpirole (D1[Gq]/D2) being significantly greater than that of animals treated with saline (p<0.001), quinpirole (p<0.001) and SKF83959 (p<0.001) alone, suggesting a supra-additive drug interaction. In contrast, the response to a combination of SKF83822+quinpirole (D1[Gs]/D2) was not significantly different from additive doses of either agonist alone.

Stereotypy (Fig 2B) following combined agonist treatment also showed effects of age (F(1,171) = 33.238, p<0.001), drug (F(5,171) = 18.668, p<0.001), and an interaction of age with drug (F(5,171) = 7.258, p<0.001). As with locomotion, adolescent stereotypy was influenced by drug (F(5,69) = 5.438, p<0.001), but only quinpirole increased stereotypy relative to saline (p<0.001). Adult stereotypy was also significantly influenced by drug (F(5,102) = 23.321, p<0.001). In adults, however, the combination of SKF83959+quinpirole (D1[Gq]/D2) increased stereotypy relative to saline (p<0.001), SKF83959 (p<0.001), and quinpirole alone (p<0.001), suggesting a supra-additive potentiation. Similarly, the stereotypic response to the combination of SKF83822+quinpirole (D1[Gs]/D2) was significantly higher than that of saline (p<0.001), SKF83822 (p = 0.001), or quinpirole (p = 0.005) alone, suggesting supra-additive D1[Gs]/D2 interactions.

### Regional IEG Expression

**Fig 3:**
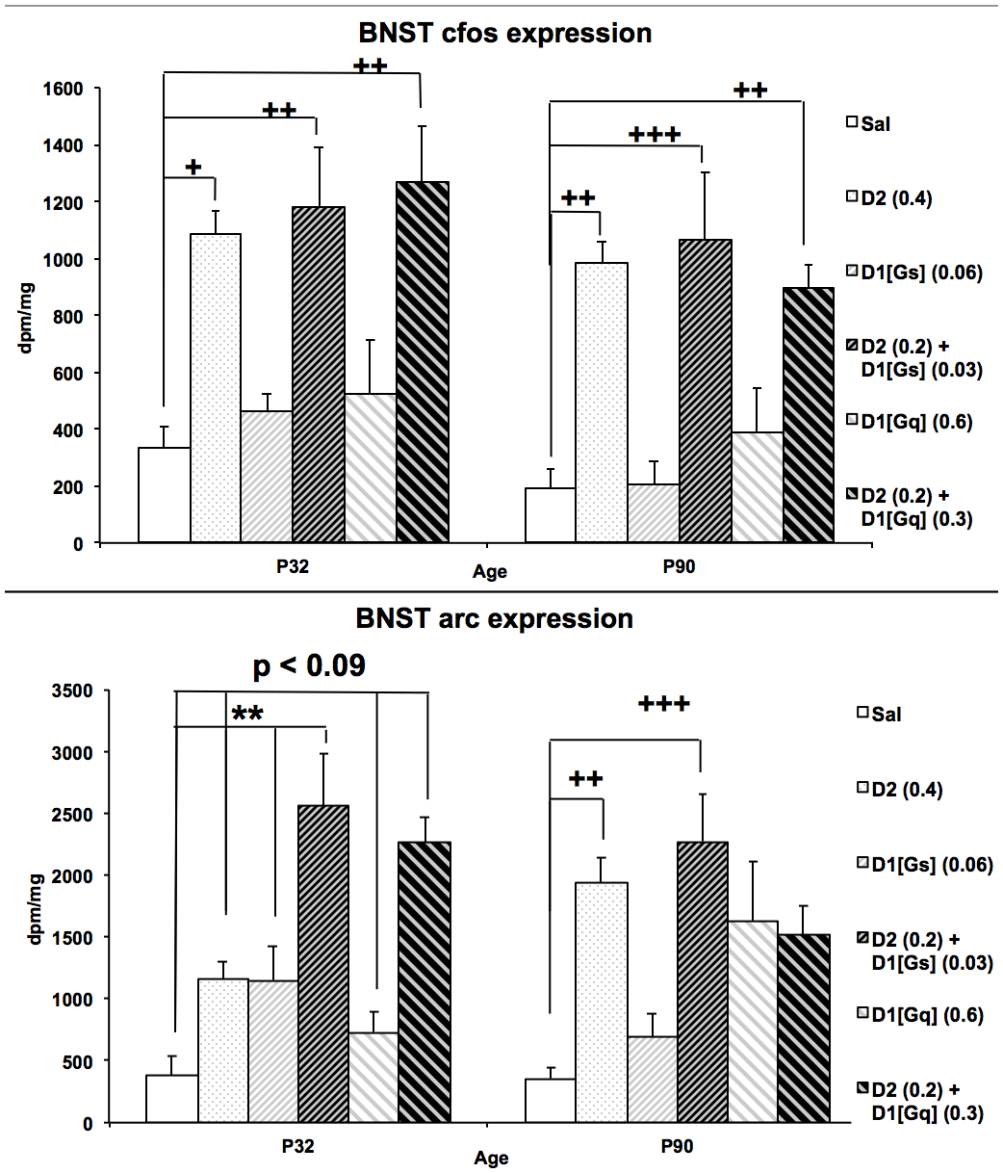
Age Differences in BNST IEG Synergy. **(A)** *Cfos* expression in the BNST of animals treated with saline, agonist alone, or in combination **(B)** *Arc* expression in the BNST of animals treated with saline, agonist alone, or in combination ***p<0.001 vs. saline and additive doses of each agonist along at the same age. +p<0.05 vs. saline, ++p<0.01; n = 6–7 per group

Given the considerable age differences in locomotor responses to D1/D2 agonists, both activity (*cfos*) and plasticity-related (*arc*) gene expression was assessed in behaviorally tested animals (S1 and S2 Tables). The most robust regional drug and age effects were observed in nuclei of the extended amygdala and stress system, particularly the BNST (Fig 3). BNST *cfos* expression was sensitive to age (F(1,61) = 5.089, p = 0.028), with greater BNST *cfos* expression in adults compared to adolescents. There was not a significant age x drug interaction (F(5,61) = 0.280, P = 0.923), and both ages showed similar robust drug effects (adolescents (F(5,31) = 7.415, p < 0.001); adults (F(5,30) = 9.363, p < 0.001)). At both ages, D2 agonism alone (adolescent p = 0.024; adult p = 0.003) and in combination with both D1[Gs] (adolescent p = 0.08, adult p = 0.001) and D1[Gq] agonists (adolescent p = 0.002, adult p = 0.010) increased *cfos* expression relative to saline controls. BNST *arc* expression was sensitive to both drug (F(5,59) = 14.187, p < 0.001) and the interaction of drug with age (F(5,59) = 2.958, p = 0.019). Although agonists increased *arc* expression in both adolescents (F(5,29) = 11.863, p < 0.001) and adults (F(5,30) = 6.280, p < 0.001), supra-additive D1/D2 interactions were observed in adolescents only. The BNST serves as an integration point linking cortical, hippocampal, and striatal circuitry to the extended amygdala and stress-sensitive nuclei [47]. It receives a multitude of anatomically and neurochemically distinct afferents, and has similar efferent heterogeneity [48], making it a densely complex nucleus whose influences at the circuit level can be difficult to predict, at times producing divergent responses depending on which subpopulations of neurons are activated [49]. Thus, activation measured at the resolution of *in situ* hybridization cannot be assumed to reflect identical influences on behaviorally relevant neural networks. Given the complex integrative role of the BNST and the importance of network dynamics in producing behavioral output [50], we undertook a data-driven exploratory assessment of coordinated neural activity via adaptation of functional network analysis to IEG data to examine the effects of dopaminergic stimulation on functional networks in adolescents and adults.

### Age Differences in Baseline CGE Networks

**Fig 4:**
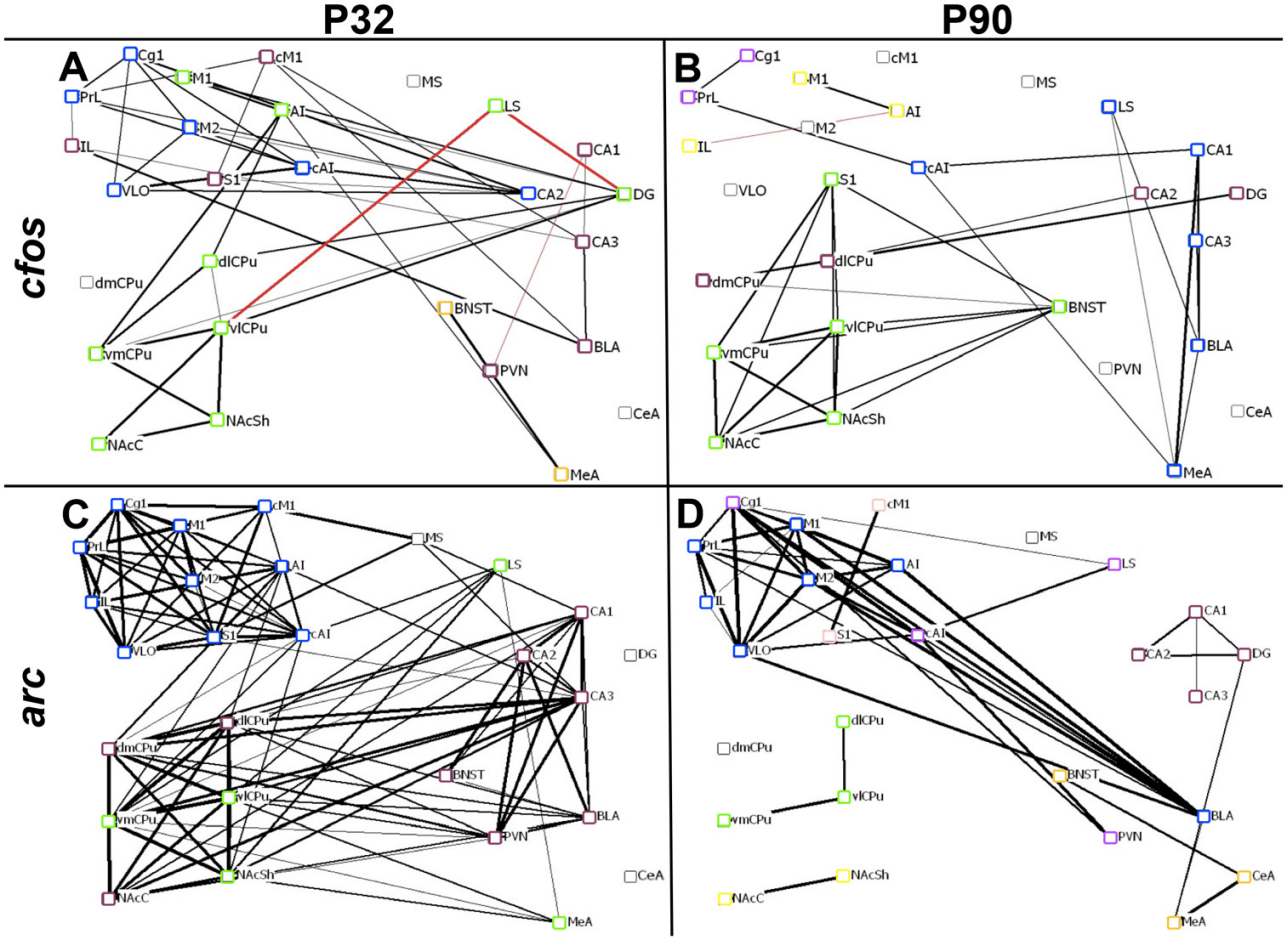
Baseline *Cfos* and *Arc* Networks. Nodes represent brain regions and are presented in pseudoanatomical space. Edges are lines connecting two nodes, and represent significant r values (p<0.05, n = 6–7), in which thicker lines correspond to more robust r values. Positive and negative r values are depicted with black and red lines, respectively. Colors outlining nodes denote modularity, in which nodes that share the same border color participate in the same sub-community. **(A)** *Cfos* networks in saline-injected adolescent controls exhibit local coordinated gene expression (CGE) in the cortex and striatum. **(B)** Adult baseline *cfos* networks are sparser than adolescent networks, and functional communities are comprised of more anatomically distributed nuclei. **(C)** Adolescent baseline *arc* networks are characterized by functional communities predominately comprised of nuclei that are anatomically proximal to each other, particularly in the cortex. **(D)** Adult baseline *arc* networks show a smaller number of functional interrelationships than adolescent networks, but demonstrate unique corticoaccumbens CGE.

To examine age differences in functional network architecture, regional networks of *cfos* and *arc* mRNA expression in saline-treated animals were constructed (Fig 4). Control *cfos* CGE maps exhibited marked organizational differences between adolescents and adults, with age-dependent community structure (colors outlining nodes denote communities). Saline-treated adolescents (Fig 4A) had a greater overall number of functional relationships than adults (Fig 4B), congruent with human imaging studies suggesting that functional connectivity becomes less diffuse and more efficient in adulthood [51]. Although both adolescents and adults show intrastriatal *cfos* CGE in baseline conditions, extrastriatal functional relationships are age-specific.

*Arc* network interactions were distinct from those of *cfos*, but shared some features of developmental transitions. Adolescents (Fig 4C) showed more coordinated *arc* expression both within brain areas (cortex, hippocampus, striatum), and across brain regions, which may reflect enhanced synchronized functional network plasticity in the developing brain. As with *cfos*, *arc* expression in the cortex was highly coordinated in adolescents, transitioning to more distributed cortical functional relationships in the adult (Fig 4D). Similarly, more functional relationships in *arc* networks were lost than gained in the transition from adolescence to adulthood. An exception, however, was coordinated *arc* mRNA expression between BLA and cortex, which matured post-adolescence.

### Age Differences in D1/D2 Agonist-Mediated CGE Networks

#### Cfos

**Fig 5:**
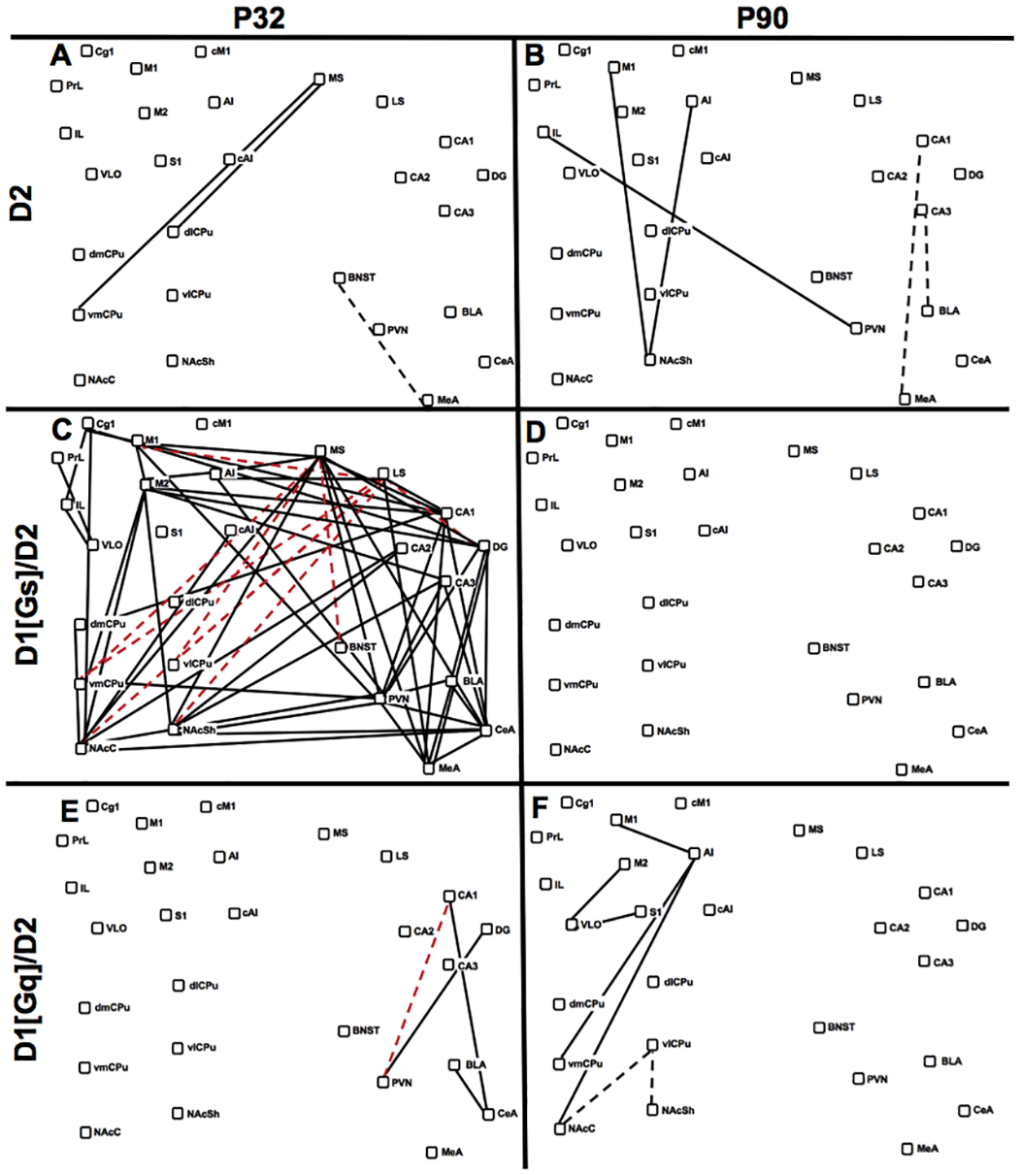
D1/D2-Induced *Cfos* Network Changes. Difference maps visually represent significant differences between baseline networks in saline-injected controls and that in animals injected with D1/D2 drug combinations. Nodes represent brain regions and are presented in pseudoanatomical space. Lines indicate functional relationships between nodes that are significantly altered by D1/D2 treatment relative to controls (p<0.01, n = 6–7). Solid black lines indicate gain of a positive *cfos* CGE. Dashed black lines indicate loss of positive *cfos* CGE. Solid red lines indicate gain of negative *cfos* CGE. Dashed red lines indicate loss of negative CGE. **(A)** Effects of D2 stimulation in adolescents. **(B)** Effects of D2 stimulation in adults. **(C)** D1[Gs]/D2 combination treatment in adolescents. **(D)** Effects of D1[Gs]/D2 combination treatment in adults. **(E)** Effects of D1[Gq]/D2 combination treatment in adolescents. **(F)** Effects of D1[Gq]/D2 combination treatment in adults.

Drug-induced changes in CGE were assessed by comparing drug-activated networks to baseline networks at each age. Adolescents treated with quinpirole, who showed robust locomotor activity, had enhanced septostriatal CGE and loss of positive CGE within the stress system (BNST, MeA) (Fig 5A). D1[Gs]/D2-treated adolescents showed little locomotor behavior, but had the most extensive network-level enhancements, with increased *cfos* CGE within the cortex, between sensorimotor cortex and striatum, between amygdala/hypothalamus and the hippocampus, and between the hippocampus and striatum (Fig 5C). There was also loss of negative interactions between the MS/LS and striatal nuclei. Changes in adolescent *cfos* network activity resulting from D1[Gq]/D2 treatment (Fig 5E) were a small subset of those induced by D1[Gs]/D2, and were focused on increased CGE between hippocampus and hypothalamus (PVN) and amygdala (CeA, BLA). In contrast, adult animals showed a loss of hippocampal network interactions following D2 agonist treatment, which was not evident with either D1/D2 combination. In adults, corticostriatal relationships were increased by D2 alone (Fig 5B) and the D1[Gq]/D2 combination (Fig 5F). Despite shared corticostriatal enhancement, the PVN was selectively integrated into networks of quinpirole-treated adults, who showed low levels of horizontal activity. Taken together, *cfos* CGE networks illustrated age differences in D1/D2 regulation of network architecture, with dopaminergic regulation of hippocampal interrelationships in adolescence and enhancement of corticostriatal relationships in adults. Drug treatments producing low behavioral output were associated with incorporation of stress nuclei into *cfos* CGE networks at both ages.

#### Arc

**Fig 6:**
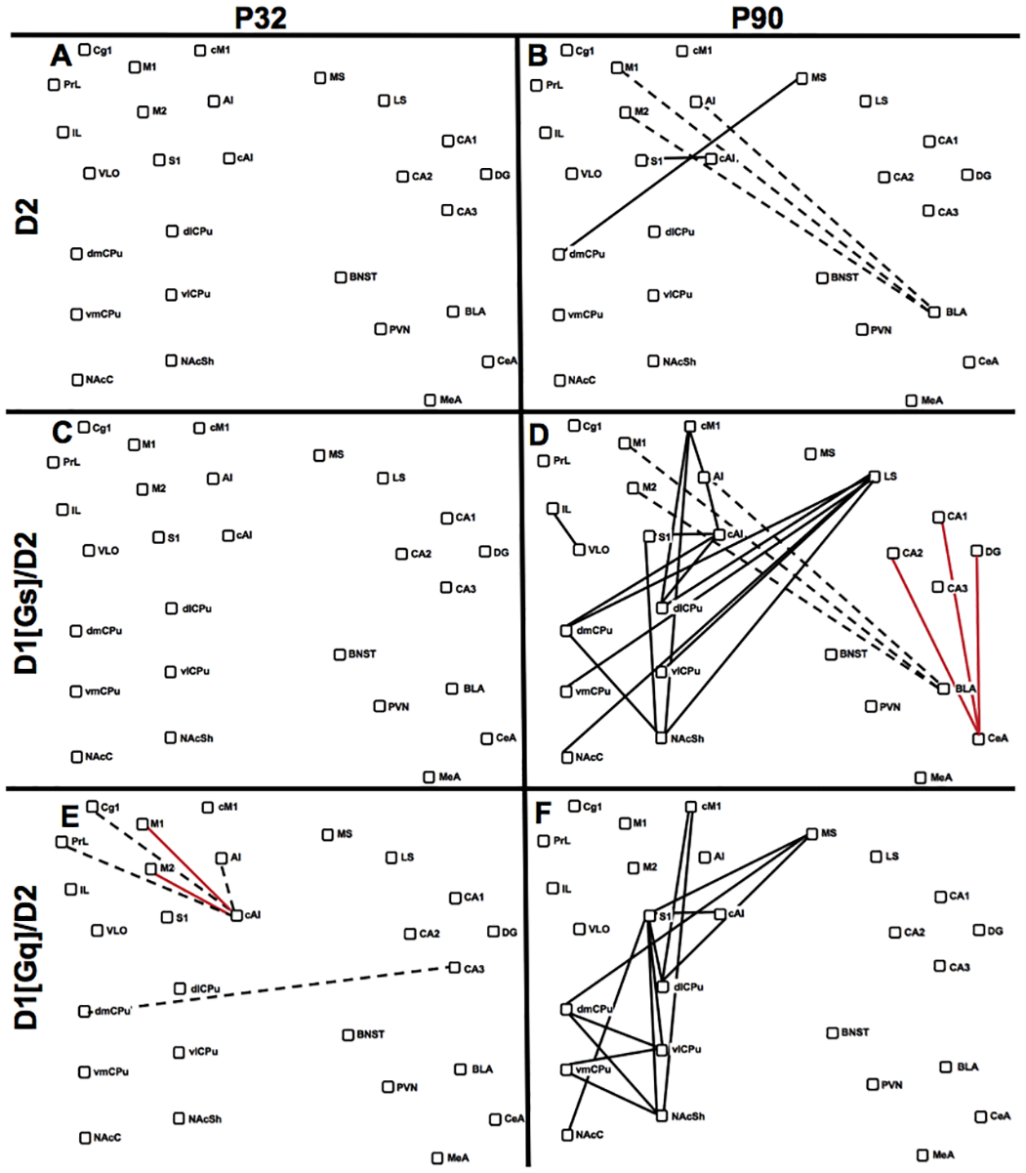
D1/D2-Induced *Arc* Network Changes. Difference maps visually represent significant differences between baseline networks in saline-injected controls and that in animals injected with D1/D2 drug combinations. Nodes represent brain regions and are presented in pseudoanatomical space. Lines indicate functional relationships between nodes that are significantly altered by D1/D2 treatment relative to controls (p<0.01, n = 6–7). Solid black lines indicate gain of a positive *arc* CGE. Dashed black lines indicate loss of positive *arc* CGE. Solid red lines indicate gain of negative *arc* CGE. Dashed red lines indicate loss of negative CGE. **(A)** Effects of D2 stimulation in adolescents. **(B)** Effects of D2 stimulation in adults. **(C)** D1[Gs]/D2 combination treatment in adolescents. **(D)** Effects of D1[Gs]/D2 combination treatment in adults. **(E)** Effects of D1[Gq]/D2 combination treatment in adolescents. **(F)** Effects of D1[Gq]/D2 combination treatment in adults.

Network analysis of *arc* expression in animals treated with D1/D2 agonist combinations revealed both age-dependent and second-messenger selective modulation of plasticity-related CGE (Fig 6). Despite robust effects of D1[Gs]/D2 treatment on adolescent *cfos* CGE, this drug combination did not significantly alter adolescent *arc* networks (Fig 6C). D1[Gq]/D2 treatment, in contrast, markedly disrupted the positively coordinated cortical *arc* expression seen in adolescent controls and introduced some negative interrelationships (Fig 5E). In adults, dopaminergic regulation of *arc* networks was also second-messenger specific. D1[Gs]/D2 treatment increased *arc* CGE within the cortex and between LS and striatal regions (Fig 5D). D1[Gs]/D2 also disrupted the highly correlated *arc* expression between BLA and cortex seen in adult controls (Fig 5D), as seen with D2 alone (Fig 5B), and induced a negative functional relationship between the CeA and hippocampus. In contrast, D1[Gq]/D2 treatment significantly increased corticostriatal, intracortical, and intrastriatal *arc* CGE (Fig 5F), without any effect on the tightly correlated BLA-cortex functional relationship. Taken together, dopaminergic modulation of adult *arc* CGE was highly second-messenger dependent with negative amygdalocortical regulation by D2 and D1[Gs]/D2 and positive corticostriatal regulation by D1[Gq]/D2. In contrast, adolescents showed minimal impact of D2 and D1[Gs]/D2 stimulation on *arc* networks, but significant reduction of intracortical *arc* CGE by D1[Gq]/D2.

## Discussion

These data demonstrate that the functional consequences of D1 and D2 receptor activation are immature during adolescence, and that D1[Gs] and D1[Gq] receptor interactions with D2 receptors are unique in both adolescents and adults. Behaviorally, quinpirole was more efficacious in adolescence, but its interactions with D1 agonists did not demonstrate the supra-additive D1/D2 interactions shown in adults, potentially underlying the blunted behavioral responses to indirect agonists that have been reported [12,13]. In contrast, the D1[Gs] agonist, SKF83822, was more effective in adults and interacted supra-additively with quinpirole to potentiate stereotypy, which may suggest synergistic interactions. The D1[Gq] agonist, SKF83959, induced little to no behavior alone, but robustly potentiated behavior when combined with quinpirole to induce locomotion and stereotypy in adults, consistent with prior descriptions of D1/D2 behavioral synergy [41,52–54]. Several of these treatments robustly activated *cfos* and *arc* expression in the BNST, whose integrative function prompted an exploratory network-level analysis of CGE. Age differences in CGE networks in the drug-free state paralleled that of functional connectivity in human imaging studies [4], progressing from local and diffuse adolescent networks to distributed and efficient adult networks. CGE patterns also illustrated age- and second messenger-specific D1/D2 regulation of network architecture.

### Immature Behavioral D1/D2 Interactions During Adolescence

Our data confirm prior findings that quinpirole is more efficacious in stimulating locomotion and stereotypy in adolescents than adults [25,26]. While quinpirole is non-selective within the D2-like family, quinpirole-stimulated locomotion in adolescence is sensitive to the D2 selective antagonist L-741,626, but not to a D3 or D4 selective antagonist [55]. Despite prior suggestions that D1 receptors are functionally underdeveloped in adolescence [26], this is the first report using second messenger selective agonists to confirm that D1[Gs]-linked stimulation induces greater behavioral response in adults. These findings suggest a developmental shift in DA regulation of ambulatory behavior from predominant control by D2 receptors in adolescence to D1[Gs] receptors in adults. While there are no available pharmacokinetic data comparing adolescent and adult metabolism of these experimental compounds, each drug significantly increased stereotypy at both ages (Fig 1) with similar timecourses (S4 Fig), suggesting significant CNS penetration. While subtle age differences in pharmacokinetic profiles cannot be excluded, significant pharmacodynamic age differences between adolescents and adults are supported by age differences in receptor expression [56], as well as age-specific effects of D1/D2 activation on neural circuit physiology [57], locomotor behaviors (Fig 2), and functional networks (Figs 5 and 6).

In adults, combined D1[Gs]/D2 stimulation supra-additively increased stereotypy, but not locomotion, whereas D1[Gq]/D2 supra-additively increased both behaviors. Thus, while D1[Gq] activation alone fails to stimulate locomotor responses, it may more readily potentiate behavior when combined with D2 agonism, consistent with predictions from molecular studies [19]. In contrast, adolescents show a fundamental difference in D1/D2 interactions, lacking supra-additive potentiation of locomotion and stereotypy following both D1[Gs]/D2 or D1[Gq]/D2 treatment when tested at these low dose combinations. Since powering an ideal synergism study using multiple D1/D2 drug combinations at differing points of efficacy to conduct isobolgraphic statistical analysis [35] would be challenging, a simplified approach to examining D1/D2 interactions was taken here [36]. This approach aims to demonstrate positive functional interactions by showing that combinations of half-doses of ineffective agonist concentrations (i.e. those ineffective at stimulating locomotor behavior based on Experiment 1) produce a significant response when additive doses alone do not. The interpretation of these supra-additive interactions as synergistic is most straightforward for adult D1[Gq]/D2-stimulated locomotion, where neither agonist alone generates ambulation at any dose tested (Fig 1A and 1E), but significant behavior is produced from the combination, similar to prior descriptions of requisite behavioral D1/D2 synergy [41,52–54]. For adult stereotypy, in which all agonists can produce significant behavior alone, isobolographic analysis would be needed to statistically confirm these interactions as synergistic. The interpretation is further complicated in adolescents, who not only fail to show the supra-additive effects assessed in this paradigm, but rather appear to exhibit negative functional interactions. The between-group experimental design and testing of only single D1/D2 combinations limits the statistical analysis of negative functional interactions, however antagonistic D1/D2 interactions in younger animals have been reported previously [30,31]. Future studies utilizing multiple direct agonist combinations to allow isobolographic statistical analysis of D1/D2 interactions are needed [35] to fully demonstrate the antagonistic and synergistic interactions that have been suggested in adolescents and adults, respectively. It has been reported that D1 and D2 receptors cannot be co-immunoprecipitated in the striatum of young animals, suggesting that the proposed, and somewhat controversial, D1[Gq]/D2 heterooligomer is late maturing [19]. This lack of signalplex formation may provide a mechanism for the lack of supra-additive behavioral D1[Gq]/D2 interactions in adolescence, but does not readily provide an explanation for the negative interactions, suggesting that circuit-level mechanisms may be at play. Furthermore, although D1[Gs] receptors are thought to signal more traditionally in the striatum and not form functional signaling complexes with D2 receptors [19], we demonstrate significant behavioral interactions between D1[Gs] and D2 agonists in adults. Thus, some of the behavioral interactions between D1 and D2 agonists that we have observed likely reflect interaction at a circuit or network level rather than the molecular level.

To begin to assess the neural circuitry underlying these behaviors, saline and drug-stimulated *cfos* and *arc* expression was examined. As this behavioral paradigm focused on low-dose agonist interactions, low regional drug-induced IEG expression was expected, a limitation of the current design. Further studies using higher dose D1/D2 combinations are needed to demonstrate the ontogeny of regional corticostriatal IEG expression, as we have previously described for the indirect agonist cocaine [15]. While low-dose agonist-induced corticostriatal IEG was minimal, robust regional drug effects were demonstrated within stress-sensitive nuclei. The BNST, which functions to integrate corticostriatal circuitry with hippocampal, amygdala, and stress networks [47] is activated by psychostimulants [58] and by combined direct D1/D2 stimulation (Fig 3). While both ages showed activation of this nucleus following D1/D2 stimulation, there were supra-additive D1/D2 interactions only in adolescents. This significant activation of the BNST by D1/D2 drug combinations in adolescence may contribute to the inhibited locomotor response seen at this age, as this nucleus is implicated in freezing behavior [59]. However, activation of the BNST in behaviorally tested animals was not associated with decreased behavioral output in all groups, and the cytoarchitectural diversity within this small nucleus makes it difficult to ascertain whether similar neuronal subpopulations are engaged across groups. Additionally, D1 and D2 receptor expression within this complex architecture is similarly complicated, and may be translationally regulated by stimuli like stress and dopamine release [60], making it difficult to predict the impacts that receptor stimulation may have at the circuit and behavioral levels. Since the BNST is well-positioned to influence network behavior and subtle differences in subregional activation can produce widely varying circuit dynamics [49], exploratory network analysis was undertaken to determine whether the observed behavioral synergistic and antagonistic interactions were associated with unique patterns of functional connectivity between brain regions.

### Correlated Interregional IEG Expression Is Influenced by Age and Drug

Graph theoretical methods have been used to characterize and quantitate features of complex networks, and have been applied to both structural and functional systems in the brain [44]. Fundamental to this analysis is the assumption that brain regions comprising functional processing networks will have highly correlated neuronal activities [61] and, thus, functional connectivity reflects patterns of deviations from statistical independence between brain regions [62]. As there are diverse methodologies to measure regional brain activation, analysis of functional brain networks has been applied to numerous human imaging paradigms (fMRI [63], magnetoencephalography [64], electroencephalography [65] and PET [66]). There have been far fewer studies examining functional connectivity in animals, although a recent study using rodent fMRI suggests that resting networks in the rat share similar properties to those in humans [67].

Neuronal activation in rodents has been measured using IEG expression, particularly *cfos* activation, for many years [68,69] and autoradiography provides exquisite spatial resolution compared to fMRI. Thus, functional network approaches are readily suitable to analyze IEG data in rodents. An additional benefit of applying network approaches to IEG analysis is that genes with functions related to plasticity can highlight networks of nuclei that may undergo coordinated synaptic modification. Whether drug exposure during adolescence alters developmental plasticity-related gene programs is a particularly important clinical question. Thus, we analyzed CGE of *cfos* mRNA as a high-resolution readout of functional associations in metabolic activity [68,69] and CGE of *arc* mRNA to illustrate plasticity-related functional networks [70,71].

The present study is one of the first to adapt these network methods to examine CGE of *cfos* and *arc* mRNA expression and to examine developmental changes. As with resting human functional connectivity networks [4,72,73], networks of *cfos* expression in rodent forebrain transitioned from local, diffuse adolescent CGE to distributed, efficient adult networks. Adolescent brain also exhibited strong *arc* CGE both within and between brain areas. Given the development of long-range structural [74] and functional relationships [73] in the transition to adulthood, high *arc* CGE is consistent with enhanced plasticity in the adolescent brain. While adults showed reduced overall *arc* CGE, coordination between the BLA and cortical nuclei was uniquely present in adulthood, potentially mediated by the late-maturing anatomical connection [74].

Complex, age-specific effects of D1/D2 agonists on CGE were observed. Although network comparisons in the present study are limited by power, group differences were observed even when using moderate correction by false discovery rate. Appropriate analysis to balance mitigation of Type I errors from multiple comparisons while avoiding the Type II errors inherent with conservative thresholds, particularly in low signal-to-noise networks, is an area of active debate [46]. The present study places an emphasis on avoiding Type II errors in this data-driven analytic approach. Testing of the hypotheses generated from these network studies with more conservative thresholding is currently underway.

While adolescent locomotor responses to combined D1/D2 stimulation were low, *cfos* CGE was substantially altered, particularly hippocampal relationships with hypothalamus and amygdala. Whereas adolescents treated with quinpirole alone showed only increased septostriatal *cfos* CGE and decreased CGE within the extended amygdala (Fig 5A), combination with either D1 agonist recruited stress nuclei to functional networks and was associated with low locomotor and stereotypic behavior. The hypothesis that adolescent locomotor behavior may be inhibited by D1/D2 activation of stress networks requires further testing. Adolescent *arc* networks were disrupted by DA agonists, with the D1[Gq]/D2 combination inducing striking dysregulation of cortical *arc* CGE. The impact of network disruption from acute or repeated adolescent drug exposure is not known, and future studies should address how these modifications occur over time using complementary longitudinal imaging approaches (e.g. fMRI) guided by predictions from high-resolution plasticity-related gene network analyses.

D1 and D2 agonists also modulated adult functional networks, with positive regulation of *cfos* CGE in corticostriatal circuitry that has been traditionally associated with DA-mediated behaviors [75]. Whereas quinpirole enhanced corticostriatal *cfos* CGE, it also recruited stress components into its network (Fig 5B). While adult D1/D2 activated networks showed second messenger specificity, neither treatment recruited components of the extended amygdala or stress system (Fig 5). Thus, as with adolescents, recruitment of stress circuitry into adult functional networks was associated with reduced locomotion. Plasticity-related networks also exhibited age-specific DA regulation, with *arc* CGE in the late-maturing BLA-cortex showing second-messenger specificity. While tightly correlated in control animals, BLA-cortical *arc* CGE was disrupted by quinpirole, alone and in combination with D1[Gs], but not with D1[Gq]. In contrast, D1[Gq]/D2 increased *arc* CGE not only within the striatum, but also in corticostriatal and hippocampo-striatal circuitry. Thus, D1[Gq]/D2 induced the most robust behavioral synergy in adults, and showed unique enhancement of corticostriatal functional relationships, both at the level of immediate activation (*cfos*) and of plasticity-related gene expression (*arc*). It is noteworthy that adolescents treated repeatedly with indirect agonists do not exhibit locomotor sensitization [14,76] (although see [12,77]), which relies on plasticity of corticostriatal circuits [75,78]. Thus, enhancement of corticostriatal coordinated plasticity-related gene expression by D1[Gq]/D2, but not D1[Gs]/D2, may be predicted to induce locomotor sensitization. Although this hypothesis has not yet been tested explicitly, recent molecular studies provide support, showing that D1[Gq]/D2 but not D1[Gs]/D2 stimulation induces striatal BDNF expression [79], a critical step in the development of behavioral sensitization [80].

While these data suggest marked differences between adolescent and adult animals, they do not necessarily imply linear development of DA-mediated behaviors and modulation of functional networks. Indeed, some data suggest that juvenile animals resemble adults in several aspects of brain and behavior, with adolescents presenting as distinct from both younger and older animals [9]. The way in which adolescence is defined as a developmental period continues to evolve, undergoing refinement or expansion as new developmental data is acquired [10,11]. The animals in this study were tested prior to puberty, in what is generally considered early adolescence [9]. Thus, as the present study does not fully describe the ontogeny of these behavioral and network phenomena, future studies should incorporate a wider range of ages including early, mid, and late adolescence in order to thoroughly characterize the developmental trajectory. Another limitation of the present study is that the stress of animal shipment may have differential effects on animals transported at younger ages versus in adulthood. Early life stress impacts the development of a number of critical brain circuits, including the dopamine system [81] and the developing hippocampus [82]. Thus, future studies could better assess the status of animal stress responses (e.g. corticosterone measurement) or avoid this confound altogether by breeding animals on site to eliminate shipping stress. An additional interpretive caveat regards the complex question of cause and effect relationships between gene expression and behavior, and the risk of implying that regional gene expression and functional networks are necessarily the drivers of behavior, rather than effects arising from the behaviors themselves. While the present studies demonstrate network-behavior associations, future studies could more mechanistically expand upon these findings by probing the causative roles of neuronal activation. For example, driving BNST neurons optogenetically could clarify if this nucleus plays developmentally distinct roles in modulating locomotor behaviors and functional networks. Lastly, these data imply an important role for stress sensitive systems in adolescence, despite the fact that stress was not specifically manipulated in these studies. Thus, future studies incorporating stress as an independent variable are warranted to more thoroughly characterize its relevance to DA-regulated behavioral networks.

### Conclusions

Taken together, these data suggest that supra-additive behavioral D1/D2 interactions may be late maturing, and that functional and plasticity-related neural networks show age differences in their modulation by dopaminergic agonists. The BNST may function as an integrative switch, recruiting stress networks inhibitory to locomotor behaviors following D1/D2 stimulation in adolescents, but not adults, a hypothesis requiring further testing. IEG network analysis suggests that normal plasticity-related CGE is disrupted by D1/D2 stimulation in adolescence, with implications for the long-term effects of indirect agonists that are routinely administered clinically in this young population. In contrast, combined D1[Gq]/D2 stimulation produces greater behavioral activation than D1[Gs]/D2 in adults, which is associated with enhanced coordination of both activity- and plasticity-related gene expression in corticostriatal circuitry. Although novel, network methods using IEG data provide a high-resolution complement to analysis of traditional imaging techniques. Comparison of network development across species should allow greater translation of studies in rodents to humans, with potential for experimental psychopharmacology and validation of animal disease models.

## Supporting Information

S1 Fig Example of Correlation Matrix. (TIF)

S2 Fig Example of Thresholded Correlation Matrix. (TIF)

S3 Fig Pseudoanatomical Regional Layout for Network Figures. Cingulate cortex (Cg1), prelimbic cortex (PrL), infralimbic cortex (IL), ventrolateral/orbital cortex (VLO), primary motor cortex (M1), secondary motor cortex (M2), primary sensory cortex (S1), caudal primary motor cortex (cM1), agranular insular cortex (AI), caudal agranular insular cortex (cAI), dorsomedial caudate putamen (dmCPu), dorsolateral caudate putamen (dlCPu), ventromedial caudate putamen (vmCPu), ventrolateral caudate putamen (vlCPu), nucleus accumbens core (NAcC), nucleus accumbens shell (NAcSh), CA1 of hippocampus (CA1), CA2 of hippocampus (CA2), CA3 of hippocampus (CA3), dentate gyrus (DG), medial septum (MS), lateral septum (LS), bed nucleus of the stria terminalis (BNST), paraventricular nucleus of the hypothalamus (PVN), basolateral amygdala (BLA), central nucleus of the amygdala (CeA), medial nucleus of the amygdala (MeA).
(TIF)

S4 Fig Examples of stereotypy timecourses following injections of D1 or D2 agonists. While there are significant effects of time (F(5,38) = 37.5, p<0.001) and time x drug (F(10,78) = 3.2, p = 0.002), there is no significant effect of time x age (F(5,38) = 0.578, p = 0.72).
(JPG)

S1 File This supplement provides a detailed description of the following methods. (I) behavioral testing procedures, (II) in situ hybridization conditions, (III) quantitative autoradiography analysis of immediate early gene expression, (IV) a step-by-step guide to the implementation of Coordinated Gene Expression (CGE) Analysis, the adaptation of functional network analysis to immediate early gene data, and (V) a step-by-step guide to statistically comparing CGE networks.
(DOC)

S1 Table *Cfos* regional gene expression. N = 6–7; +p < 0.05 vs. saline, ++p<0.01, +++p<0.01, (+)p<0.09
(TIF)

S2 Table *Arc* regional gene expression. N = 6–7; +p < 0.05 vs. saline, ++p<0.01, +++p<0.01; **p<0.01 vs saline, additive doses of each agonist alone at same age, (*)p<0.09
(TIF)
