# Supplementary figures and images for: Adolescent Maturation of Dopamine D1 and D2 Receptor Function and Interactions in Rodents
Source: PLoS One. 2016 Jan 19;11(1):e0146966. doi: 10.1371/journal.pone.0146966 (PMC4718668; doi:10.1371/journal.pone.0146966)

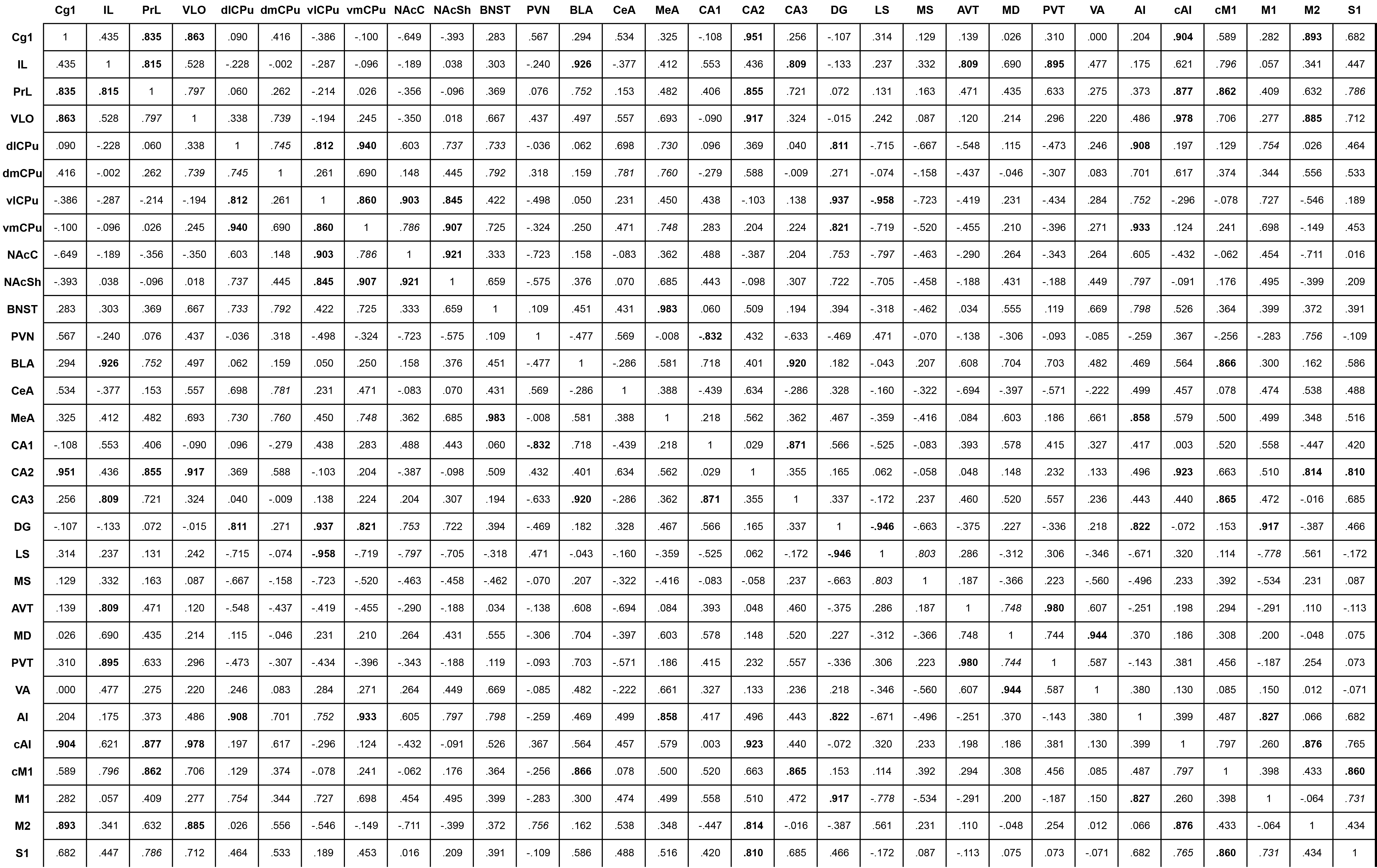

Supplement: S1 Fig — (TIF) [file pone.0146966.s001.tif]

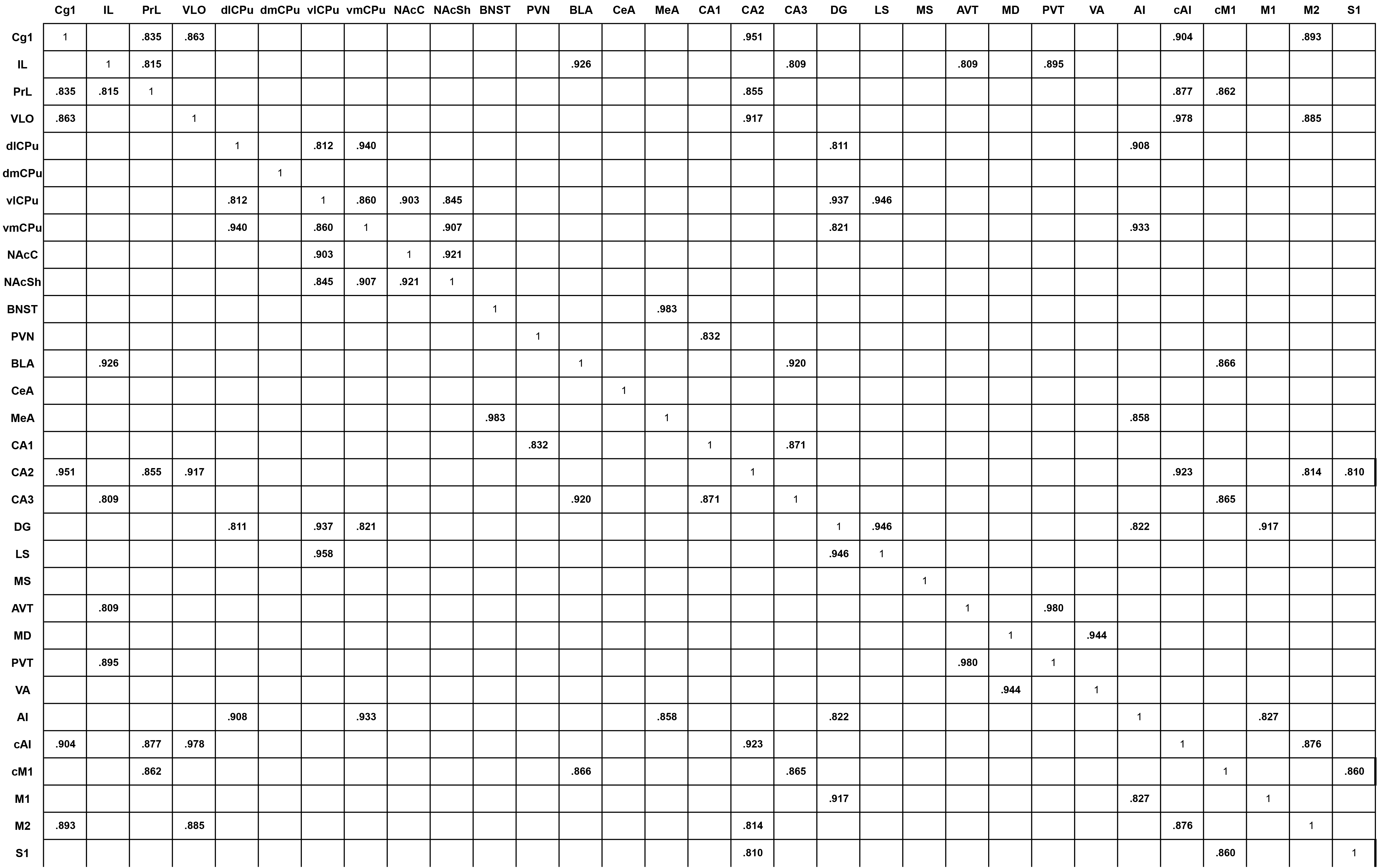

Supplement: S2 Fig — (TIF) [file pone.0146966.s002.tif]

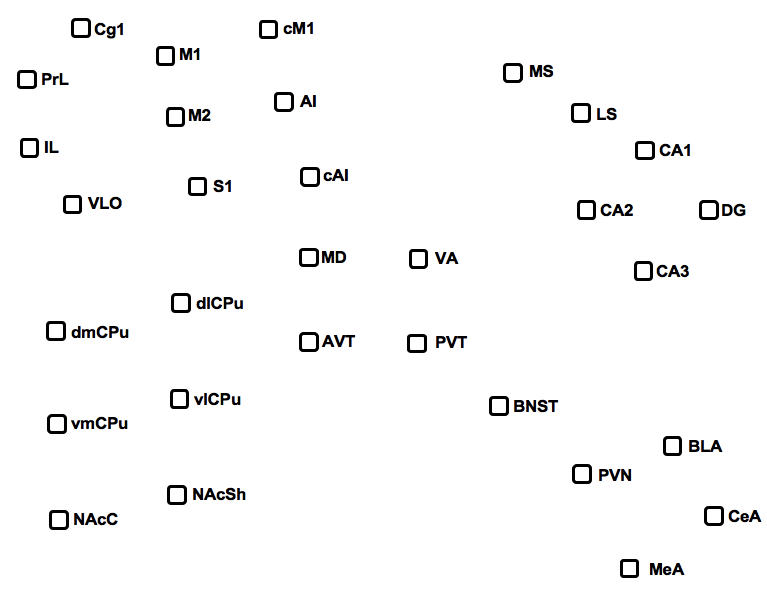

Supplement: S3 Fig — Cingulate cortex (Cg1), prelimbic cortex (PrL), infralimbic cortex (IL), ventrolateral/orbital cortex (VLO), primary motor cortex (M1), secondary motor cortex (M2), primary sensory cortex (S1), caudal primary motor cortex (cM1), agranular insular cortex (AI), caudal agranular insular cortex (cAI), dorsomedial caudate putamen (dmCPu), dorsolateral caudate putamen (dlCPu), ventromedial caudate putamen (vmCPu), ventrolateral caudate putamen (vlCPu), nucleus accumbens core (NAcC), nucleus accumbens shell (NAcSh), CA1 of hippocampus (CA1), CA2 of hippocampus (CA2), CA3 of hippocampus (CA3), dentate gyrus (DG), medial septum (MS), lateral septum (LS), bed nucleus of the stria terminalis (BNST), paraventricular nucleus of the hypothalamus (PVN), basolateral amygdala (BLA), central nucleus of the amygdala (CeA), medial nucleus of the amygdala (MeA). (TIF) [file pone.0146966.s003.tif]

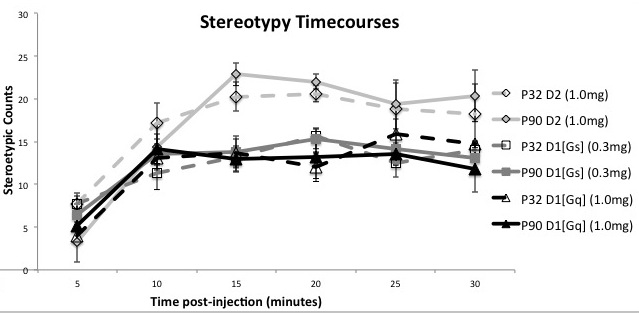

Supplement: S4 Fig — While there are significant effects of time (F(5,38) = 37.5, p<0.001) and time x drug (F(10,78) = 3.2, p = 0.002), there is no significant effect of time x age (F(5,38) = 0.578, p = 0.72). (JPG) [file pone.0146966.s004.jpg]

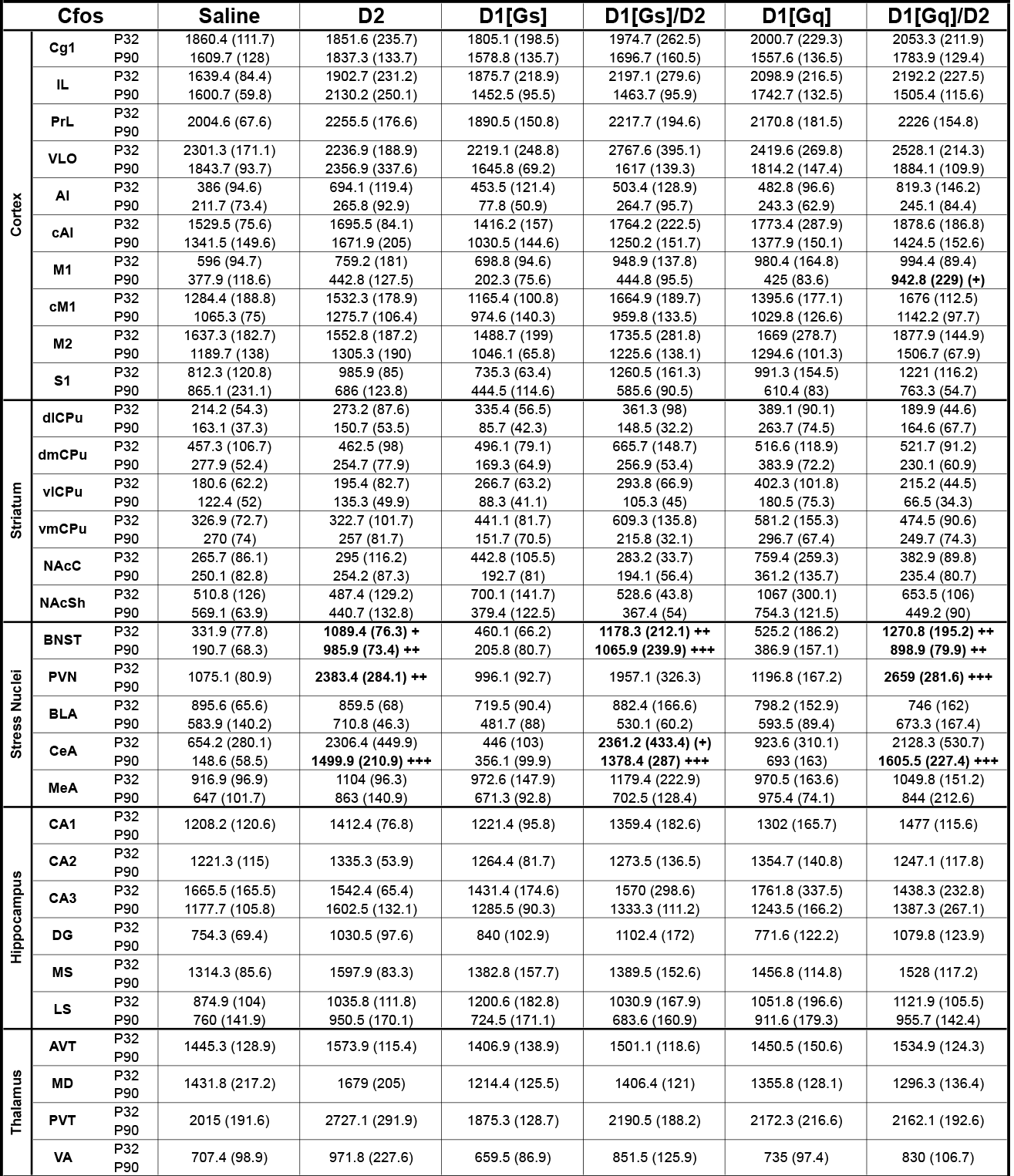

Supplement: S1 Table — N = 6–7; +p < 0.05 vs. saline, ++p<0.01, +++p<0.01, (+)p<0.09 (TIF) [file pone.0146966.s006.tif]

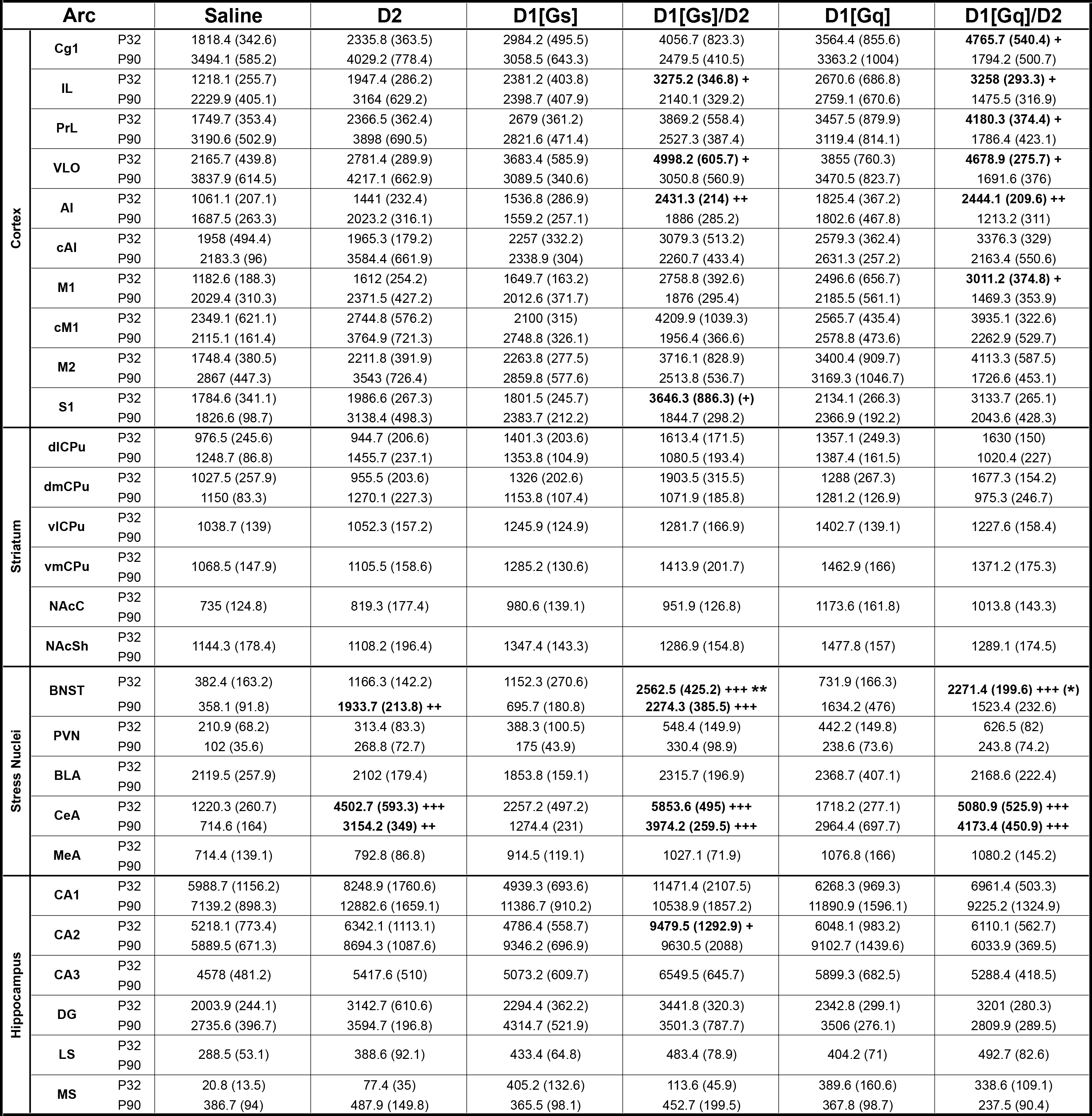

Supplement: S2 Table — N = 6–7; +p < 0.05 vs. saline, ++p<0.01, +++p<0.01; **p<0.01 vs saline, additive doses of each agonist alone at same age, (*)p<0.09 (TIF) [file pone.0146966.s007.tif]
