# Supplementary material for: Adolescent Maturation of Dopamine D1 and D2 Receptor Function and Interactions in Rodents
Source: PLoS One. 2016 Jan 19;11(1):e0146966. doi: 10.1371/journal.pone.0146966 (PMC4718668; doi:10.1371/journal.pone.0146966)
Supplement: S1 File — (I) behavioral testing procedures, (II) in situ hybridization conditions, (III) quantitative autoradiography analysis of immediate early gene expression, (IV) a step-by-step guide to the implementation of Coordinated Gene Expression (CGE) Analysis, the adaptation of functional network analysis to immediate early gene data, and (V) a step-by-step guide to statistically comparing CGE networks. (DOC) [file pone.0146966.s005.doc]

**Supplemental Methods:**

I. Behavioral Methods

All behavioral testing was conducted using four identical open-field activity systems (Med Associates, St. Albans, VT) measuring 43.2cm x 43.2 cm x 30.5cm. Horizontal locomotion was recorded by 16 evenly spaced infrared monitors located on two adjacent sides of the chamber, with infrared box size appropriately adjusted for the size difference between adolescents and adults. As beam breaks are translated into ambulatory counts, it is important to tailor infrared box sizes to the relative sizes of the animals (i.e. so that small adolescent animals do not need to travel a further distance to record an ambulatory count), as has been described previously (McQuown et al., 2009). On test day, rats were placed into the novel locomotor apparatus for a 30min habituation period. Following habituation, rats were injected i.p. with saline or drug and locomotor behavior was recorded for the next 30 min. Concurrently, a blinded observer scored stereotypic behavior for 10sec of every minute using a modified version of the scale of LaHoste and Marshall (1992): where 0=inactivity, 1=grooming, 2=locomotion, 3=sniffing up, 4=sniffing down, 5=sniffing down in a circumscribed area, 6=sniffing down and licking the floor, 7=licking or gnawing the test apparatus, 8=self biting. Grooming, sniffing, and locomotion can all be performed in stereotypic or non-stereotypic ways. In these studies only stereotyped grooming and sniffing were scored as such, however to truly determine confirm stereotyped locomotion, involving locomotion along a repatitive route, a sampling time greater than 10 sec per minute would be needed. Thus, cumulative stereotypy is labed as total “stereotypy rating” in the figures. In experiments in which animals were sacrificed following behavioral testing, rapid decapitation occurred 30 minutes post-drug injection.

II. In Situ Hybridization

Coronal sections were cryostat cut at -20°C at a thickness of 20μm. Sections were mounted onto poly L-lysine-coated glass slides and fixed with 4% paraformaldehyde in 0.1M phosphate-buffered saline (PBS) for 1h at room temperature, then washed in 0.1M PBS for 3 x 5 min, dried, and stored in airtight boxes at -20°C until use.

Tissue sections were processed for in situ hybridization according to the method of Winzer-Serhan et al (1999). For c-fos, [^35^S]-labeled riboprobes were transcribed in antisense and sense directions from a pGEM-3Z plasmid containing a 680bp fragment of c-fos cDNA between T7 and SP6 promoter sites (kindly provided by Dr Stanley Watson, University of Michigan). For arc, [^35^S]-labeled riboprobes were transcribed in antisense and sense directions from a pBSII-SK+ containing 3032bps of arc cDNA between T7 and T3 promotor sites (kindly provided by Dr. Paul Worley, Johns Hopkins University). Sections were pretreated with proteinase K (0.05μg/ml), acetylated, dehydrated through graded ethanols (50, 75, 95, and 100%), and air dried, then incubated for 18h at 60°C with hybridization solution containing ^35^S -labeled sense or antisense riboprobes (1 x 10^7^ cpm/ml). After hybridization, sections were treated with RNase A and washed at high stringency. Tissue sections were dehydrated and exposed to Kodak Biomax film for 1 day with ^14^C standards of known radioactivity.

III. Quantitative Analysis of Autoradiograms

Autoradiographic images were quantified using a computer-based image analysis system (MCID, Image Research Inc., St Catharines, ON, Canada). Brain areas on autoradiograms were defined by comparison of Nissl stained sections with the atlas of Paxinos and Watson (1998). Optical densities in discrete brain regions were measured and the corresponding values of radioactivity were determined by interpolation from a standard curve, generated from ^14^C standards of known radioactivity (Broide et al. 1995). In each brain region, mRNA expression was quantified by subtracting corresponding regional measures of sense hybridization. mRNA expression was expressed as dpm/mg wet weight. Regional averages were obtained from readings of the right and left hemispheres from two comparable sections for each brain region.

IV. Step-by-Step Guide to Coordinated Gene Expression (CGE) Analysis

1. Obtain values of regional mRNA expression through traditional methods of quantitative autoradiography (see section II Quantitative Analysis of Autoradiograms, above).
2. Each age and drug treatment must be analyzed separately. Calculate intersubject Pearson correlation coefficients (r) between each pair of brain regions analyzed using the values of gene expression levels derived from the quantitative mRNA expression analysis (Step 1, above). For example, the values of Cg1 versus IL cfos mRNA expression from every saline-treated adolescent are used to calculate an r value for Cg1-IL in this group. An r value is derived for every pair of brain regions analyzed. Pearson correlation coefficients can be calculated via SPSS software (SPSS 17.0, Chicago, IL) and imported in Microsoft Excel. These r values can be displayed as a symmetrical matrix with values ranging from -1 to +1 (S1 Fig), with a separate matrix for each age and treatment group.
3. Choose a threshold for the matrices in order to visualize the networks. The threshold for the present data was set to r values equating to a p value of ≤ 0.05. While choosing the values at which to threshold matrices for visualization purposes has been a topic of active discussion (Rubinov and Sporns, 2010), a p value of 0.05 was chosen in these data sets as it is a standard, albeit arbitrary, choice in large array of statistical analyses. While the threshold at which relationships are truly functionally relevant within a given brain circuit is unknown, using the standard p≤0.05 allows us to focus on the tightest functional relationships, which are arguably the most likely to be functionally relevant. For visualization purposes, all r-values below that threshold are set to 0, yielding a thresholded matrix (S2 Fig). Note that the diagonal dividing the center of the matrix consists of reflexive connections (r = 1), which are ignored.
4. Visualize the matrix using network software, such as UCINET and Netdraw (UCINET 6.0, Analytic Technologies, Lexington, KY). Each weighted, undirected, thresholded network must be imported into UCINET software. UCINET matricies can then be visualized in Netdraw software. The visualization renders each brain region that labels the axes of the matrix as a network node, shown as labeled squares (S3 Fig). The nodes are displayed in pseudanatomical space, in which nodes representing subregions of the same larger brain area are clustered together (e.g. subregions of prefrontal cortex are grouped together in the upper left hand corner). R-values above the predetermined threshold are displayed as edges, or lines connecting 2 nodes to each other. Black lines denote positive r values and red lines denote negative r values. Since these matrices are weighted rather than binary, the thickness of the line denotes the strength of the r-value. Thus, the values closest to +1 or -1 will be represented by the thickest black and red lines, respectively.
5. Visualization of Community Structure. As the use of functional network analysis increases, shared resources have allowed more in depth analysis of network structure. The open-source brain connectivity toolbox (Rubinov and Sporns, 2010) contains several programs that analyze network matricies using Matlab ((Matlab R2010a, MathWorks, Natick, MA). For these data, the modularity function was used (modularity_und.m), which separates networks into optimized community structure, in which subcommunities are more highly connected to nodes within their groups than to nodes outside of their groups. The output of this function assigns each node to an individual community. Nodes that belong to the same functional communities are denoted in the network visualizations using node outline color. This feature aids in highlighting the groups of regions that may be anatomically distant, but are highly functionally integrated.

V. Step-by-Step Guide to CGE Network Comparison

1. Full, unthresholded matrices (those described in Section III, Step 2, above) should be used to compare networks to each other. While a certain r value may not reach the significant criteria chosen for visualization, drug treatment may significantly alter that value compared saline networks.
2. Transform r values to Z scores using the Fischer transformation ( ½[ln(1 + r) – ln(1 – r)]). As the distribution of r values tends to be skewed from a normal distribution, the r-to-Z transformation improves normality for more accurate comparison. Thus, the step produces a matrix of identical size as the original matrix, save for containing Z scores rather than r values for each relationship.
3. Calculate the difference of Z scores by subtracting the saline condition from the drug condition for each pairwise comparison. E.g. If Saline Cg-IL Z-score is 0.50 (derived from r value of 0.46) and D1[Gq]/D2 Cg-Il Z-score is 0.82 (derived from r value of 0.68), the difference of Z scores would be 0.82 – 0.50 = 0.32.
4. Divide the difference of Z scores by the pooled standard error of the Z score, calculated as SE_z_ = Sqrt[(1/(n_1_ – 3)) + (1/(n_2_ – 3))], in which n is the number of subjects included in the correlation analysis.
5. Take the absolute value of the calculated Z values obtained in Step 4 to determine the p value equivalent. Be sure to set p value criteria for 2-tailed test in order to detect both increases and decreases relative to baseline. These data were corrected via false discovery rate with a liberal threshold of q=0.35. This threshold hold was chosen with the priority of minimizing Type II errors, while still providing some correction for Type I error from multiple comparisons.
6. To visualize difference networks, proceed in the same fashion used to visualize networks described in Section III, Steps 3 and 4. That is, remove all values below the chosen threshold and import the thresholded matrix in the UCINET and netdraw.

S1 Table. *Cfos* regional gene expression. N= 6-7; +p < 0.05 vs. saline, ++p<0.01, +++p<0.01, (+)p<0.09

S2 Table. *Arc* regional gene expression. N= 6-7; +p < 0.05 vs. saline, ++p<0.01, +++p<0.01; **p<0.01 vs saline, additive doses of each agonist alone at same age, (*)p<0.09

S1 Fig. Example of Correlation Matrix.

S2 Fig. Example of Thresholded Correlation Matrix

S3 Fig. Pseudoanatomical Regional Layout for Network Figures. Cingulate cortex (Cg1), prelimbic cortex (PrL), infralimbic cortex (IL), ventrolateral/orbital cortex (VLO), primary motor cortex (M1), secondary motor cortex (M2), primary sensory cortex (S1), caudal primary motor cortex (cM1), agranular insular cortex (AI), caudal agranular insular cortex (cAI), dorsomedial caudate putamen (dmCPu), dorsolateral caudate putamen (dlCPu), ventromedial caudate putamen (vmCPu), ventrolateral caudate putamen (vlCPu), nucleus accumbens core (NAcC), nucleus accumbens shell (NAcSh), CA1 of hippocampus (CA1), CA2 of hippocampus (CA2), CA3 of hippocampus (CA3), dentate gyrus (DG), medial septum (MS), lateral septum (LS), bed nucleus of the stria terminalis (BNST), paraventricular nucleus of the hypothalamus (PVN), basolateral amygdala (BLA), central nucleus of the amygdala (CeA), medial nucleus of the amygdala (MeA).

S4 Fig: Examples of stereotypy timecourses following injections of D1 or D2 agonists. While there are significant effects of time (F(5,38)=37.5, p<0.001) and time x drug (F(10,78)=3.2, p=0.002), there is no significant effect of time x age (F(5,38)=0.578, p = 0.72).
